# Stuck Outside and Inside: An Exploratory Study on the Effects of the COVID-19 Outbreak on Italian Parents and Children’s Internalizing Symptoms

**DOI:** 10.3389/fpsyg.2020.586074

**Published:** 2020-10-22

**Authors:** Cristiano Crescentini, Susanna Feruglio, Alessio Matiz, Andrea Paschetto, Enrico Vidal, Paola Cogo, Franco Fabbro

**Affiliations:** ^1^Department of Languages and Literatures, Communication, Education and Society, University of Udine, Udine, Italy; ^2^Department of Psychology, Sapienza University of Rome, Rome, Italy; ^3^Division of Pediatrics, Department of Medicine (DAME), University of Udine, Udine, Italy

**Keywords:** Covid-19, internalizing symptoms, parents, children, resilience

## Abstract

The Covid-19 outbreak and the subsequent lockdown have profoundly impacted families’ daily life, challenging their psychological resilience. Our study aimed to investigate the immediate psychological consequences of the pandemic on Italian parents and children focusing on internalizing and post-traumatic symptoms. We also wanted to explore the impact of possible risk and resilience factors, e.g., lifestyle and behaviors, emotional and cognitive beliefs, on parents and children’s reaction to the emergency distress. An online survey was administered during the country’s nationwide lockdown to 721 Italian parents of at least one child aged between 6 and 18 years. The respondent completed the survey for himself/herself and his/her child. The survey included socio-demographic items and validated questionnaires on parents’ post-traumatic stress symptoms, depression and anxiety levels, and on children’s internalizing problems. Parents were asked to fill the questionnaires twice: once referring to the current emergency condition and once recalling how they and their child felt a few months before Covid-19 outbreak. Multiple regression analyses showed that specific demographic characteristics (i.e., sex and age) and psychological factors of children and parents, such as fear of contagion and the opportunity to think about possible secondary positive effects of the pandemic, had a predictive value on the presence of internalizing symptoms of both parents and children. Moreover, parents’ behaviors during the lockdown period (i.e., employment status and sport practiced) were significantly related to their own internalizing symptoms; these symptoms, in turn, had a strong and positive predictive value on children’s internalizing problems. Besides, analyses of variance showed that internalizing symptoms of parents and children were significantly higher during the Covid-19 pandemic than before it started. In addition to showing a direct effect of the pandemic on the psychological health of parents and children, the present results also give a series of important information on how parents perceive, and therefore influence, their children in this period of emergency. Our findings thus highlight the urgent need to provide parents with adequate support to take care of their own psychological wellbeing and to help their children coping with the direct and indirect effects of the pandemic.

## Introduction

The outbreak of the novel coronavirus SARS-Cov2 has led to a global health emergency with alarming implications, not only for individual and collective health, but also for emotional and social functioning (Dubey et al., 2020; Pfefferbaum and North, 2020). Children may be among the most exposed to the psychosocial consequences of the pandemic due to a major disruption in their daily life, and their immature ability to process the short- and long-term effects of the emergency. A better understanding of how children’s psychological wellbeing has been affected and, more generally, how the family system has been impacted is required to find out protective and risk factors associated with mental health during the Covid-19 outbreak, and to deliver adequate support to parents and children in need.

As confirmed cases approached 110,000 patients across over 100 countries, the Covid-19 outbreak has been declared a pandemic by the World Health Organization (World Health Organization [WHO], 2020, Situation Report-51, 11th March 2020). One country after another adopted strict measures to limit the spread of the viral pneumonia, such as physical-distancing, and temporary closure of schools, universities and non-essential workplaces. Many governments indeed ordered a nationwide lockdown limiting movements of the entire population: people could not leave their home, except for a proven state of emergency or necessity. Italy was the first country in Europe to report a significant number of infections and to adopt restrictive measures. Schools and universities were closed in the worst affected regions of northern Italy in late February and, at the beginning of March, Italian prime minister announced a government decree imposing a nationwide lockdown (DPCM, 9th March 2020 in Gazzettaufficiale, 2020). The outbreak and the consequent lockdown had a profound economic and social impact and, as current literature is revealing, they significantly affected the mental health of general population: subsyndromal mental health concerns, such as depressive and anxiety symptoms, seem to be a common response to the pandemic (Rajkumar, 2020; Wang et al., 2020).

Monitoring the effects of Covid-19 outbreak across high-risk groups has become a priority and young people are likely to be among the most affected by the psychosocial consequences of the emergency (Fegert et al., 2020; Holmes et al., 2020). During Covid-19 outbreak, children’s routine was drastically disrupted due to the closure of schools and lack of outdoor activities, resulting in limited connection with classmates and friends, absence of a day-to-day schedule, and increased sedentary behaviors and screen time (Xiang et al., 2020). Furthermore, children’s immature ability to understand and process what was happening during and in the aftermath of the emergency made them even more vulnerable (Balaban, 2006). These arguments were supported by a preliminary study conducted in China (the first country where the epidemic developed), that reported the presence of psychological difficulties in children aged 3–18 years during the pandemic, with clinging, inattention, irritability and worries as the most severe symptoms (Jiao et al., 2020). Besides, during past epidemic diseases (i.e., N1H1, SARS, and Asian influenza), a high percentage of children who were isolated or quarantined developed acute stress and adjustment disorders: parents reported that nearly one-third of children who were quarantined met the clinical criteria for post-traumatic stress disorder (Sprang and Silman, 2013).

Overall, children exposed to emergencies and disasters can exhibit several negative psychological outcomes: they may develop internalizing problems as anxiety-related symptoms, e.g., excessive worries and fears, and depressive symptoms, e.g., becoming detached and numb, or somatic complaints, e.g., headache and stomachache (Balaban, 2006; Danese et al., 2020). It is important to bear in mind that most of these symptoms are transient, can be considered an expected reaction to intense distress, and may not require immediate clinical intervention (Danese et al., 2020). Nonetheless, we can assume that many children, during and after Covid-19 pandemic, may need special support and reassurance from their parents, as well as appropriate and simple information to understand what is happening, and they should be monitored to identify and prevent the possible development of more severe long-lasting disorders.

The direct engagement of children in systematic screening and assessment in the context of traumatic and distressing experiences should be preferred; though, if children cannot be observed directly, a widely used mean to assess the presence of children’s behavioral and emotional symptoms is to ask caregivers to evaluate them, for example using the Child Behavioral Checklist Parent Report Form (Achenbach, 1991; Achenbach and Rescorla, 2001; Balaban, 2006). Although well validated, this method has been debated as parents’ reports of their children’s problems might be biased by their own psychopathology and by the sex of the child (Najman et al., 2001; Kroes et al., 2003). When possible, the mental health status of caregivers should be evaluated at the same time as children: many studies have shown that parental adjustments during emergencies are important predictors of children’s mental health outcomes (McFarlane et al., 1987; Laor et al., 2001); moreover, one of the greatest risk factors for children to develop a psychopathology is having a parent with a psychiatric disorder (see e.g., Beidel and Turner, 1997; Maciejewski et al., 2018). Both genetic and environmental factors seem to be involved in the familiar transmission of psychological disorders, but the exact nature of the underlying mechanisms remains still unclear.

During Covid-19 pandemic and the prolonged home confinement imposed, it is possible that children’s problems may have been exacerbated by their parents’ stress. Suddenly, most parents had to rearrange their schedule and find a new balance between their personal life, smart working organization, and children’s management. This situation put them under great pressure, and the most vulnerable parents may have become too overwhelmed to find appropriate ways to be supportive caregivers and to address children’s fears and insecurities, increasing the risk of children experiencing behavioral and emotional problems (Spinelli et al., 2020). Interestingly, during a past epidemic, Remmerswaal and Muris (2011) found that parents and children’s fear of being infected were significantly correlated, and parents’ fear was associated with the transmission of threat information to their offspring, which in turn was linked to children’s fear of the disease (the link remained significant even when controlling for other sources of information, i.e., media, friends, school, or direct experience with the disease). Thus, parents could have a great influence on children’s wellbeing in this period of emergency, and taking into account the whole family system becomes essential.

The main aim of the present study was to investigate the immediate psychological effects of Covid-19 pandemic and the consequent lockdown on children, as reported by their parents, and on parents themselves. We focused on internalizing and post-traumatic stress symptoms, controlling for those demographic factors that are most associated with their incidence (i.e., age and gender; Altemus et al., 2014). We expected that internalizing problems (i.e., behavioral and emotional problems, often occurring concurrently, with prominent anxiety, withdrawal, depressive and somatic symptoms unexplained by medical conditions; Achenbach et al., 2016) might have increased (with respect to normative data) during the health emergency in both parents and children (see e.g., Jiao et al., 2020; Wang et al., 2020) and that parents’ difficulties might have had a negative impact on children’s wellbeing (see e.g., Spinelli et al., 2020). Furthermore, little is known about which factors may be associated with parents and children’s mental health during a health emergency. Therefore, we aimed at exploring the impact of possible risk and protective, resilience factors on parents and children’s reaction to the emergency distress: such as lifestyle and behaviors (i.e., the amount of sport practiced by the parents, parents’ employment status, and the number of children’s close friends), and emotional and cognitive beliefs (i.e., parents’ fear of being infected by SARS-Cov2 and parents’ ability to broaden their biased attention on the pandemic crisis by thinking about its possible secondary positive effects or implications). Finally, parents and children’s current wellbeing could have been partly influenced by their prior condition, thus we were also interested in incorporating a retrospective research design (i.e., asking parent participants to report on the basis of their memories). We thus asked parents to rate their own anxiety and depression problems, and those of their children, twice: once referring to the current emergency period, and once recalling how they and their children felt before Covid-19 outbreak.

## Materials and Methods

### Participants and Procedure

An online survey among parents of, at least, one child aged between 6 and 18 years living in northern or central Italy was administered from April 16 to May 07, 2020, during the country’s nationwide lockdown. A member of the parenting couple completed the survey for himself/herself and his/her child. The first part of the survey included a socio-demographic questionnaire (40 items) focused on how parents and their children were experiencing the health emergency. Next, participants completed 3 validated questionnaires on impact of events, depression and anxiety levels, and the internalizing problems of their children (i.e., symptoms of anxiety, depression and somatic complaints). Parents were asked to fill the questionnaires twice: once referring to the current health emergency (a condition called Cov) and once recalling how they and their children felt the months before Covid-19 outbreak, namely the last months of 2019 (a condition called PreCov). To help participants in the PreCov condition, the survey instructions explained to them that a useful way to remember how they (and their children) felt a few months earlier, could be to observe the photos of that period that the participants could have kept on their mobile phones. The order of presentation of the questionnaires was counterbalanced across participants (i.e., there were two possible sequences: Cov, PreCov or PreCov, Cov). The impact of event scale was only filled once with reference to the current health emergency.

Participants were initially recruited using word-of-mouth and through contacting school leaders and school teachers; the questionnaires were initially equally distributed in the two sequence orders. Then, participants were also recruited by snowball sampling overall resulting in 849 respondents though not perfectly counterbalanced in terms of the two questionnaires’ sequence orders (see Table 1). Thus, we decided to take the sequence order factor into consideration in the data analysis. All data were collected using Google Forms. The procedures were approved by the local Ethics of the University of Udine and were in accordance with the Helsinki Declaration guidelines. All participants provided informed consent.

**TABLE 1:** Raw scores of participants.

	IES-R_Cov	HADS_Cov anxiety	HADS_Cov depression	CBCL_Cov anxiety	CBCL_Cov withdrawn/depressed	CBCL_Cov somatic complaints
Whole sample of respondents (age 42.80 years ± 5.47) *N* = 721	23.16 ± 15.71	6.06 ± 3.70	5.49 ± 3.67	5.14 ± 3.95	2.81 ± 2.84	1.58 ± 2.01
Sex_Parent (‘0’) = Female 85.71%	24.68 ± 15.90	6.38 ± 3.74	5.76 ± 3.68	5.31 ± 3.97	2.94 ± 2.87	1.71 ± 2.08
Sex_Parent (‘1’) = Male 14.29%	14.02 ± 10.66	4.14 ± 2.80	3.88 ± 2.20	4.12 ± 3.64	2.02 ± 2.47	0.81 ± 1.13
Sex_Child (‘0’) = Female 48.40%	23.87 ± 15.78	6.12 ± 3.75	5.61 ± 3.67	5.21 ± 3.90	2.69 ± 2.99	1.78 ± 2.10
Sex_Child (‘1’) = Male 51.60% (age whole sample: 10.07 years ± 2.52)	22.49 ± 15.62	6.01 ± 3.66	5.38 ± 3.68	5.08 ± 3.99	2.92 ± 2.68	1.40 ± 1.89
Sport (‘0’) = 0–1 h/day 70.46%	24.13 ± 16.14	6.41 ± 3.74	5.91 ± 3.69	5.22 ± 3.92	2.97 ± 2.88	1.67 ± 2.07
Sport (‘1’) = >1 h/day 29.54%	20.84 ± 14.39	5.23 ± 3.47	4.48 ± 3.45	4.95 ± 4.01	2.42 ± 2.69	1.38 ± 1.81
Work (‘0’) = none/suspended 42.86%	24.12 ± 16.42	6.31 ± 3.83	5.87 ± 3.71	5.45 ± 4.09	2.88 ± 3.06	1.72 ± 2.09
Work (‘1’) = remote/on site 57.14%	22.43 ± 15.13	5.88 ± 3.59	5.20 ± 3.62	4.91 ± 3.82	2.76 ± 2.66	1.48 ± 1.93
Fear (‘0’) = none/little 84.61%	21.25 ± 14.55	5.68 ± 3.57	5.25 ± 3.68	5.02 ± 3.88	2.83 ± 2.87	1.60 ± 2.06
Fear (‘1’) = much/very much 15.39%	33.61 ± 17.67	8.18 ± 3.72	6.81 ± 3.35	5.82 ± 4.23	2.69 ± 2.65	1.51 ± 1.65
BBA_1 (‘0’) = never/sometime 48.83%	26.02 ± 16.58	6.83 ± 3.84	6.48 ± 3.76	5.36 ± 3.84	3.04 ± 2.84	1.59 ± 2.05
BBA_1 (‘1’) = often/very often 51.17%	20.43 ± 14.33	5.33 ± 3.42	4.54 ± 3.32	4.93 ± 4.04	2.59 ± 2.82	1.57 ± 1.95
BBA_2 (‘0’) = never/sometime 36.48%	26.79 ± 16.78	7.01 ± 3.83	6.58 ± 3.77	5.50 ± 3.97	3.05 ± 2.90	1.53 ± 1.89
BBA_2 (‘1’) = often/very often 63.52%	21.07 ± 14.67	5.52 ± 3.52	4.86 ± 3.47	4.93 ± 3.92	2.67 ± 2.79	1.61 ± 2.06
Sequence (‘0’) = PreCov_Cov 32.73%	22.35 ± 15.74	5.97 ± 3.76	5.40 ± 3.52	4.43 ± 3.86	2.95 ± 2.89	1.45 ± 1.88
Sequence (‘1’) Cov_PreCov 67.26%	23.55 ± 15.69	6.11 ± 3.68	5.53 ± 3.75	5.49 ± 3.94	2.74 ± 2.81	1.64 ± 2.06
Friends_Child (‘0’) = 0–2 30.51%	22.10 ± 15.51	5.98 ± 3.69	5.80 ± 3.69	5.80 ± 4.32	3.25 ± 2.92	1.64 ± 1.95
Friends_Child (‘1’) = ≥3 69.49%	23.62 ± 15.78	6.10 ± 3.71	5.35 ± 3.66	4.85 ± 3.74	2.62 ± 2.78	1.56 ± 2.03

	**IES-R_PreCov**	**HADS_PreCov anxiety**	**HADS_PreCov depression**	**CBCL_PreCov anxiety**	**CBCL_PreCov withdrawn/depressed**	**CBCL_PreCov somatic complaints**

Whole sample of respondents (age 42.80 years ± 5.47) *N* = 721	N/a	5.19 ± 3.14	3.98 ± 3.22	4.56 ± 3.71	2.23 ± 2.62	1.33 ± 1.73
Sequence (‘0’) = PreCov_Cov 32.73%	N/a	5.47 ± 3.27	4.45 ± 3.15	5.41 ± 3.84	2.73 ± 2.66	1.47 ± 1.74
Sequence (‘1’) = Cov_PreCov 67.26%	N/a	5.05 ± 3.07	3.76 ± 3.24	4.15 ± 3.57	1.98 ± 2.56	1.25 ± 1.73

**Raw scores (mean and standard deviation, SD) of questionnaires (completed with reference to the Covid-19 health emergency: Cov) for the whole sample of respondents (parents of children and adolescents aged 6–18 years who rated their own symptoms of post-traumatic stress, anxiety and depression and then rated those of their children) and by function of Sex (of parents and children), Sport, Work, Fear, Friends_Child and a “broadening of biased attention” on the pandemic crisis expressed in terms of participants’ thinking about its possible secondary positive effects for one’s life (BBA_1) and for the environment (BBA_2). Data of questionnaires filled in with reference to the period preceding the start of the Covid-19 outbreak (last months of 2019: PreCov) are reported in the bottom part of the table. N/a, not available. IES-R, Impact of Event Scale-Revised; HADS, Hospital Anxiety and Depression Scale; CBCL, Child Behavior Checklist. Sequence indicates the order with which HADS and CBCL questionnaire were compiled by parents [PreCov_Cov = Sequence (‘0’) and Cov_PreCov = Sequence (‘1’)].**

As mentioned above, 849 respondents completed the survey. After excluding parents with serious physical or psychiatric conditions (55 participants), parents of atypically developing children (53 participants), those who had been infected with Covid-19 (1 participant) or whose child was not between 6 and 18 years old (17 participants), and 2 respondents who had not completed the survey correctly, we obtained a sample of 721 healthy parents of typically developing children (mean age 42.80 ± 5.47 years; 103 males, 14.2%; mean age of children 10.08 ± 2.52 years; 372 males, 51.6%) on which we based the following analyses.

The sample was mainly composed of Italian parents (709, 98.3%), who were married (524, 72.6%); most of them had 1 or 2 children (194, 26.9 and 415, 57.6%, respectively) and had a high-school diploma or a higher education level (371, 51.4%). The majority of the sample was living in a village with <2,000 inhabitants (330, 45.8%) or a small city of 2,000–10,000 inhabitants (268, 37.2%), in a house with more than 125 sqm (364, 50.5%), and had access to a garden (633, 87.7%).

### Measures

#### Socio-Demographic Questionnaire

A socio-demographic questionnaire was developed for the purpose of this study. The first part included 13 demographic questions about participants’ age, sex, nationality, education, physical and/or psychiatric conditions, marital status, number of children, the characteristics of the place of residence, the characteristics of their house, i.e., its size and if it has an outdoor space, and the number of people with whom they were living. The second group of questions (5 items) focused on parents’ lifestyle during the last 2 weeks: their employment status, the amount of time they spent every day practicing sport (range of possible answers: 0–>2.5 h per day) and with their child (<1–>5 h per day), the number of times they left home (0–>5 times) and the amount of time spent outside (<1–>4 h). Third, parents were asked to report their direct experience with the COVID-19 infection (6 items): if they had been tested with the swab, if they were positive, if they experienced COVID-19 symptoms, how much they feared being infected (no fear – very much fear of contracting the virus) and the amount of time they spent inquiring about the pandemic in the media since COVID-19 breakdown in China on January 2020 (<1–>2 h per day). Then, there were 14 questions regarding their child and his/her lifestyle before and during the pandemic: which child they were referring to (their only child, the firstborn, etc.) and why they had chosen him/her, the child’s age, sex, nationality, grade attended, if he/she had a learning support teacher and why, if he/she had a physical or psychiatric condition, how many times per week he/she used (before the Covid-19 outbreak) to meet friends outside from school (<1–≥3 times a week), which sports he/she preferred, how many close friends he/she had (0–≥4 close friends) and the amount of time he/she spent every day with the respondent parent (<1–>5 h per day). Finally, there were 2 items that aimed at exploring if parents could broaden, in the past 2 weeks, their biased attention on the pandemic crisis by thinking about its possible secondary positive effects. This aspect was operationalized as having thought (never – very often) during the past 2 weeks of the health emergency about its possible related implications or opportunities for one’s life, i.e., giving oneself more space or slowing down the frenetic pace of life, and for the environment, i.e., reducing pollution and undertaking in the future a more environmental friendly lifestyle.

#### Impact of Event Scale-Revised

After completion of the socio-demographic questionnaire, participants filled in three questionnaires. The Italian adaptation of the IES-R (Weiss and Marmar, 1996; Craparo et al., 2013) is a 22-item self-report measure of current subjective distress in response to a specific traumatic event. It comprises three subscales representative of the major symptoms clusters of post-traumatic stress: intrusion (item example: “I thought about it even when I did not mean to”), avoidance (item example: “I tried not to think about it”), and hyper-arousal (item example: “I found myself acting or feeling like I was back at that time”). The responses are rated on a 4-point Likert scale ranging from 0 (never) to 3 (very often). In the present study the participants were asked to refer to the symptoms of distress they may had experienced during the last week regarding the emergency of COVID-19 pandemic and the consequent restrictive measures adopted by the government. Overall sample Cronbach’s alpha: IES-R = 0.93.

#### Hospital Anxiety and Depression Scale

The Italian adaptation of the HADS (Zigmond and Snaith, 1983; Costantini et al., 1999) is composed of two 7-item scales that assess emotional disturbance: one for anxiety (item example: “Worrying thoughts go through my mind”) and one for depression (item example: “I look forward with enjoyment to things”). Each item is scored from 0 to 3, so the respondent can score between 0 and 21 for either anxiety or depression, with higher scores denoting higher levels of anxiety or depression. The participants of this study were asked to fill the HADS twice: once referring to the current health emergency (taking the last 2 weeks as a time reference) (the Cov condition) and once recalling how they felt the months before Covid-19 outbreak (the PreCov condition). Overall sample Cronbach’s alpha: total HADS-Cov score = 0.87; total HADS-PreCov score = 0.84.

#### Child Behavior Checklist (6–18)

The Italian adaptation of the CBCL/6-18 (Achenbach, 1991; Achenbach and Rescorla, 2001; Frigerio, 2001) is a caregiver report form used to assess behavioral and emotional problems in children and adolescents aged 6–18 years. In the present study we used 3 syndrome scales: anxious/depressed (13 items; item example: “Cries a lot”), withdrawn/depressed (8 items; item example: “There is very little he/she enjoys”), and somatic complaints (11 items; item example: “Feels dizzy or lightheaded”). Each item is scored from 0 (not true) to 2 (very true or often true), and the sum of the scores of the 3 scales corresponds to the broader dimension of internalizing problems (higher scores denote higher internalizing problems). The participants of this study were asked to fill the CBCL/6-18 twice: once referring to their child’s problems of the past 2 weeks (the Cov condition) and once recalling their child’s problems the months before Covid-19 outbreak (the PreCov condition). Overall sample Cronbach’s alpha: total CBCL-Cov internalizing score = 0.88; total CBCL-PreCov internalizing score = 0.87.

### Data Analysis

Continuous measures were summarized reporting mean and standard deviation (SD) of raw scores for both the whole sample of respondents and separately for the two levels (‘1’ and ‘0’) of each dichotomous variable considered in the following analysis (i.e., sex of the participant, sex of his/her child, amount of sport practiced by the parent, parent’s employment status, parent’s fear of contagion, parent’s broadening of biased attention on the crisis regarding oneself and the environment, number of child’s close friends, sequence of questionnaires: Cov, PreCov or PreCov, Cov) (Table 1).

The main analyses focused on the Cov condition and concerned a series of multiple linear regression models on continuous responses reported by the participants about: (1) their own levels of current post-traumatic stress (IES-R_Cov), anxiety (HADS_Cov Anxiety) and depression (HADS_Cov Depression) and (2) their children’s levels of current internalizing symptoms, in the three components of anxiety (CBCL_Cov anxiety), depression (CBCL_Cov withdrawn/depression), and somatic complaints (CBCL_Cov somatic complaints).

Dichotomous and continuous variables were introduced in the models at one single step of computation. In particular, for each of the three models concerning parents’ stress and internalizing symptoms, we introduced: (1) four demographic variables, such as sex and age of both parents and their children (Age_Parent; Age_Child; Sex_Parent; Sex_Child); (2) two dichotomous variables concerning the amount of sport practiced each day during the past 2 weeks (Sport: 0–1 vs. >1 h per day) and the participants’ current employment status (Work: unemployed or temporarily suspended vs. remote or on-site worker); (3) three dichotomous variables concerning the psychological factors of fear of infection (Fear: none or little fear vs. much or very much fear of contracting SARS-CoV2 virus) and a “broadening of biased attention” (BBA) on the pandemic crisis, which was reflected by participants’ thinking about (never or sometimes vs. often or very often during the last 2 weeks of the health emergency) its possible secondary positive effects or implications: for one’s life (for example giving oneself more space or slowing down the frenetic pace of life: BBA_1) and for the environment (for example reducing pollution and undertaking in the future a more environmental friendly lifestyle: BBA_2). Finally, a last dichotomous variable was included in the models reflecting the Sequence with which participants had to rate their own symptoms (and those of their children) of anxiety and depression. As already mentioned, there were two possible sequences: Cov, PreCov and PreCov, Cov. The impact of event scale (IES-R) was only filled once with reference to the current health emergency but the Sequence variable was maintained in the corresponding regression model.

With regards to parents’ rate of children’s internalizing symptoms, similar regressions models were ran. In particular, for each of the three models (CBCL_Cov anxiety, CBCL_Cov withdrawn/depression, CBCL_Cov somatic complaints) the same Age_Parent, Age_Child, Sex_Parent, Sex_Child, Fear, BBA_1, BBA_2, and Sequence variables were entered. Moreover, we included parents’ total HADS_Cov scores (anxiety plus depression) and the number of close friends (Friends: 0–2 vs. at least 3), who parents reported their children had before the health emergency started. Sport and Work were excluded from the three models concerning children’s internalizing symptoms.

For each regression model, significance, coefficient of determination (*R*^2^), and model coefficients (Bs with their standard error –SE- and corresponding standardized values βs) were reported. Variance inflation factors (VIFs) were calculated to avoid multicollinearity in each regression model (i.e., VIF was considered too high if ≥5; in the analyses reported below no value exceeded 1.5). To avoid alpha-inflation the alpha-level was set to 0.01 in each regression model. Effect sizes for *R*^2^ were considered small (0.02), medium (0.13) and large (0.26) (Cohen, 1988).

As secondary analysis, we compared parents’ reported Cov and Pre_Cov anxiety and depression scores, both when they gave description of themselves and of their children (see bottom part of Table 1 for the raw data concerning the Pre_Cov condition). We ran five mixed model ANOVAs with repeated measures including anxiety, depression and somatic complaints scores as within-subject variables at two levels (Time: HADS_Cov Anxiety vs. HADS_PreCov Anxiety; HADS_Cov Depression vs. HADS_PreCov Depression; CBCL_Cov Anxiety vs. CBCL_PreCov Anxiety; CBCL_Cov Withdrawn/Depressed vs. CBCL_PreCov Withdrawn/Depressed; CBCL_Cov Somatic Complaints vs. CBCL_PreCov Somatic Complaints) and Sequence (Cov_PreCov vs. PreCov_Cov) as between-subject variable. Overall, we used a statistical significance threshold of *p* < 0.05 in all ANOVAs and we reported effect sizes as partial eta squared ($\eta_{\text{p}}^{2}$). Effect sizes were considered small (0.01), medium (0.06) and large (0.14) (Cohen, 1988; Miles and Shevlin, 2001). The overall data were analyzed with Statistica 8 (StatSoft, Inc., Tulsa, OK, United States). The data that support the findings of this study are available from the corresponding author, upon request.

## Results

### Descriptive Statistics of the Sample

Table 1 reports the raw scores of participants in all questionnaires used in the present study. Referring to the cut-off and norming groups for these questionnaires (IES-R: Creamer et al., 2003; Wang et al., 2020; HADS: Zigmond and Snaith, 1983; Costantini et al., 1999; CBCL 6-18: Achenbach, 1991; Achenbach and Rescorla, 2001; Frigerio, 2001) and considering the questionnaires filled in by parents with reference to the current Covid-19 health emergency (the Cov condition), the data showed that the mean scores for post-traumatic stress, anxiety and depression symptoms of parents and internalizing problems of children were within normal ranges when compared against normative data. Nonetheless, we found that many parents reported moderate to severe post-traumatic stress symptoms (195, 27.0%; IES-R score ≥ 33); elevated symptoms of anxiety (90, 12.4%; HADS Anxiety score ≥ 8); elevated symptoms of depression (64, 8.8%; HADS Depression score ≥ 8). As regard parents evaluation of children’s internalizing problems, it emerged that a high percentage of children showed elevated anxiety (191, 26.4%; CBCL Anxious/Depressed T score ≥ 65) and depression (175, 24.2%; CBCL Withdrawn/Depressed T score ≥ 65) and, with less incidence, somatic complaints (65, 9.0%; CBCL Somatic complaints T score ≥ 65).

The socio-demographic questionnaire revealed that 309 parents (42.8%) were unoccupied or temporarily suspended from their job during the health emergency, and 130 parents (18.0%) had never left home or went outside once in the last 2 weeks. As regards participants’ daily activities during the last 2 weeks, 213 (29.5%) reported to practice sport every day (1 or more hours per day). Finally, the vast majority (628, 87.1%) spent more than 5 h per day with their children, while, before the lockdown, only 254 parents (35.2%) reported spending that same time with their children. Of all respondents, 35 (4.8%) were tested with the Covid-19 swab and the response was negative (the only respondent who resulted positive to Covid-19 infection was excluded from the sample). Nevertheless, 12 respondents believed they had been infected by the novel coronavirus (1.6%), and 60 participants (8.3%) affirmed that, in the last weeks or at the time they were filling the survey, they have had one or more Covid-19 related symptoms, such as fever, dry cough, pain muscle, nasal congestion, sore throat, diarrhea or pneumonia. Since the Covid-19 breakdown in China on January 2020, 210 parents (29.1%) have spent more than 2 h per day reading or watching the news about the health emergency on TV, newspapers or Internet (which may suggest the importance of understanding the possible effects of repeated media consumption during the crisis; Holmes et al., 2020). 111 parents (15.3%) reported being very afraid of being infected by the novel coronavirus. Finally, more than half of the parents reported that in the last 2 weeks they could broaden their attention on the pandemic crisis to consider some possible secondary positive effects such as the chance of giving oneself more space or slowing down the frenetic pace of life (369 respondents thought about it often or very often, 51.1%), and the chance to reduce pollution and undertake a more environmental friendly lifestyle (458 respondents thought about it often or very often, 63.5%).

### Multiple Regression Analyses

**FIGURE 1:**
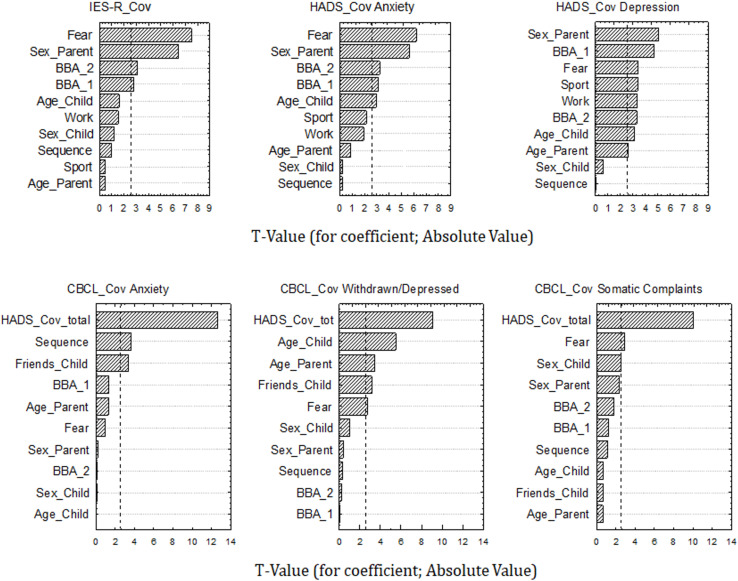
Chart of *t*-values for multiple regression coefficients. *X*-axis refers to t-values (absolute values), *y*-axis refers to variables considered in each regression model [Sex and age of Parents and Children; parents’ employment status –Work-; Fear of contagion; amount of sport practiced; the order with which HADS and CBCL questionnaire were compiled by parents: Sequence PreCov_Cov or Sequence Cov_PreCov; number of children’s close friends; parents’ total HADS_Cov score; and parents’ “broadening of biased attention” on the pandemic crisis by thinking about its possible secondary positive effects for one’s life (BBA_1) and for the environment (BBA_2)]. Vertical bar in each graph indicates significance level at p < 0.01. IES-R, Impact of Event Scale-Revised; HADS, Hospital Anxiety and Depression Scale; CBCL, Child Behavior Checklist. Cov, completion of questionnaires with reference to the Covid-19 health emergency.

In Table 2, regression analyses globally predicting post-traumatic, anxiety, depression and somatic symptoms of parents and their children (as rated by their parents) are presented. Each regression model was significant and the total variance explained generally reflected a medium effect size (Cohen, 1988; Miles and Shevlin, 2001). Figure 1 reports a schematic representation of t-values (absolute values are reported) for each multiple regression coefficient. In relation to the dependent variable IES-R_Cov, measuring parents’ symptoms of post-traumatic stress, the results showed that having high fear of being infected positively predicted IES-R_Cov scores. Reduced post-traumatic scores were instead predicted by a higher tendency to broaden biased attention on the crisis to think about its possible positive secondary effects for one’s life (BBA_1) and for the environment (BBA_2).

**TABLE 2:** Regression analyses predicting post-traumatic, anxiety, depression, and somatic symptoms in parents and children (rated by their parents).

	IES-R_Cov				HADS_Cov anxiety				HADS_Cov depression					CBCL_Cov anxiety				CBCL_Cov withdrawn/depressed				CBCL_Cov somatic complaints			
							
Variable	*B*	*SE*	β	*t*	*B*	*SE*	β	*t*	*B*	*SE*	β	*t*	Variable	*B*	*SE*	β	*t*	*B*	*SE*	β	*t*	*B*	*SE*	β	*t*

	Model: *R*^2^ = 0.18, *F*(10,710) = 15.43, *p* < 0.001				Model: *R*^2^ = 0.17, *F*(10,710) = 14.43, *p* < 0.001				Model: *R*^2^ = 0.17, *F*(10,710) = 14.71, *p* < 0.001					Model: *R*^2^ = 0.23, *F*(10,710) = 21.22, *p* < 0.001				Model: *R*^2^ = 0.16, *F*(10,710) = 13.17, *p* < 0.001				Model: *R*^2^ = 0.16, *F*(10,710) = 13.45, *p* < 0.001			
Sequence	1.13	1.16	0.034	0.97	0.06	0.27	0.01	0.21	0.01	0.27	0.01	0.03	Sequence	1.01	0.28	0.12	3.63**	–0.07	0.21	–0.01	–0.34	0.16	0.15	0.04	1.11
Sex_Parent	–10.32	1.60	–0.23	−6.46**	–2.13	0.38	–0.20	−5.63**	–1.90	0.38	–0.18	−5.07**	Sex_Parent	–0.07	0.39	–0.01	–0.18	–0.11	0.30	–0.01	–0.38	–0.50	0.21	–0.09	−2.37^+^
Age_Parent	0.05	0.11	0.02	0.47	0.02	0.02	0.03	0.91	0.07	0.03	0.10	2.61*	Age_parent	–0.03	0.03	–0.05	–1.30	–0.07	0.02	–0.13	−3.48**	0.01	0.01	0.02	0.62
Work	–1.67	1.10	–0.05	–1.52	–0.50	0.26	–0.07	–1.93	–0.86	0.26	–0.12	−3.33**	Fear	–0.35	0.37	–0.03	–0.94	–0.76	0.28	–0.10	−2.72*	–0.57	0.20	–0.10	−2.90*
Sport	–0.58	1.21	–0.02	–0.48	–0.62	0.29	–0.08	−2.15^+^	–0.96	0.29	–0.12	−3.36**	BBA_1	0.40	0.30	0.05	1.33	–0.02	0.23	–0.01	–0.07	0.20	0.16	0.05	1.25
Fear	11.19	1.49	0.26	7.51**	2.19	0.35	0.21	6.19**	1.19	0.35	0.12	3.41**	BBA_2	0.04	0.31	0.01	0.14	0.05	0.24	0.01	–0.20	0.30	0.17	0.07	1.80
BBA_1	–3.47	1.23	–0.11	−2.81*	–0.91	0.29	–0.12	−3.12*	–1.34	0.29	–0.18	−4.65**	Sex_Child	–0.03	0.26	–0.01	–0.14	0.19	0.20	0.03	0.99	–0.35	0.14	–0.09	−2.51^+^
BBA_2	–3.94	1.27	–0.12	−3.09*	–0.98	0.30	–0.13	−3.23**	–0.99	0.30	–0.13	−3.30**	Age_Child	0.01	0.06	0.01	0.06	0.23	0.04	0.21	5.48**	0.02	0.03	0.03	0.70
Sex_Child	–1.27	1.07	–0.04	–1.18	–0.05	0.25	–0.01	–0.22	–0.16	0.25	–0.02	–0.62	Friends_Child	–0.95	0.28	–0.11	−3.33**	–0.67	0.21	–0.11	−3.14*	–0.10	0.15	–0.02	–0.66
Age_Child	–0.37	0.23	–0.06	–1.60	–0.16	0.05	–0.11	−2.96*	–0.17	0.05	–0.12	−3.12*	HADS_Cov_tot	0.27	0.02	0.46	12.66**	0.14	0.02	0.35	9.10**	0.11	0.01	0.38	10.03**

^+^p < 0.05, * p < 0.01, ** p ≤ 0.001. IES-R, Impact of Event Scale-Revised; HADS, Hospital Anxiety and Depression Scale; CBCL, Child Behavior Checklist. Cov, completion of questionnaires with reference to the Covid-19 health emergency. Sequence indicates the order with which HADS and CBCL questionnaire were compiled by parents [PreCov_Cov = Sequence (‘0’) and Cov_PreCov = Sequence (‘1’)]. BBA_1 and BBA_2 refer to a “broadening of biased attention” on the pandemic crisis expressed in terms of participants’ thinking about possible its secondary positive effects for one’s life (BBA_1) and for the environment (BBA_2). BBA_1 was dichotomized with ‘Never/sometime’ as ‘0’ and ‘often/very often’ as ‘1’; BBA_2 was dichotomized with ‘Never/sometime’ as ‘0’ and ‘often/very often’ as ‘1’. The other dichotomous variables were entered as follow: Sex_Parent and Sex_Child were dichotomized with ‘Male’ as ‘1’ and ‘Female’ as ‘0’; Sport was dichotomized with ‘0–1 h/day’ as ‘0’ and ‘>1 h/day’ as ‘1’; Work was dichotomized with ‘None/suspended’ as ‘0’ and ‘remote/on site’ as ‘1’; Fear was dichotomized with ‘none/little’ as ‘0’ and ‘much/very much’ as ‘1’; Friends_Child was dichotomized with ‘0–2’ as ‘0’ and ‘≥3’ as ‘1.’

With regards to HADS_Cov anxiety, the results indicated that, similarly to IES-R_Cov scores, to be female and have high fear of contagion positively predicted parents’ anxiety scores, while BBA_1 and BBA_2 were negatively related with anxiety levels. Moreover, a negative marginal relation between the amount of sport practiced by parents in the last 2 weeks and HADS_Cov anxiety was also found.

As far as HADS_Cov depression is concerned, the results again showed the positive relations between being a female and having high fear of contagion and parents’ depression scores, as well as the negative relation between depression symptoms and BBA_1 and BBA_2. Moreover, we found that the amount of sport practiced and the parents’ employment status (on-site or remote work vs. suspension of work or unemployment) were negatively related to parents’ level of depression.

Turning to how parents rated their children’s levels of anxiety, depression and somatic complaints, the first regression model concerning CBCL_Cov Anxiety showed that parents rated their children as more anxious as they were considered to have less close friends. A strong positive relation between children’s anxiety level and parents’ total HADS_Cov scores (anxiety plus depression) was also found. Finally, an effect of Sequence was found: to have filled in the CBCL anxiety questionnaire referring first to the actual health emergency condition (CBCL_Cov Anxiety) and then considering the period before the Covid-19 outbreak (CBCL_PreCov Anxiety) positively predicted current anxiety symptoms of children as rated by their parents.

As far as CBCL_Cov Withdrawn/Depressed is concerned, the results highlighted again a strong positive relation with parents’ total HADS_Cov scores and a negative relation with children’s number of close friends. Remarkably, we found that parents’ fear of contagion negatively predicted children’s depression: to have none or little fear of being infected positively predicted children’s level of depressive symptoms as reported by their parents.

Finally, with regards to CBCL_Cov Somatic complaints, the results further showed the strong and positive predictive value of parents’ total HADS_Cov scores, as well as the negative relation with parents’ fear of being infected.

In sum, the findings of the multiple regression analyses showed that specific psychological and behavioral factors of parents and children, such as fear of contagion, the opportunity to think about possible secondary positive effects of the pandemic and the number of children’s close friends, had a predictive value on the presence of internalizing symptoms of both parents and children. Moreover, parents’ behaviors during the lockdown period were significantly related to their own internalizing symptoms; these symptoms, in turn, had a strong and positive predictive value on children’s internalizing problems.

### Analyses of Variance

**FIGURE 2:**
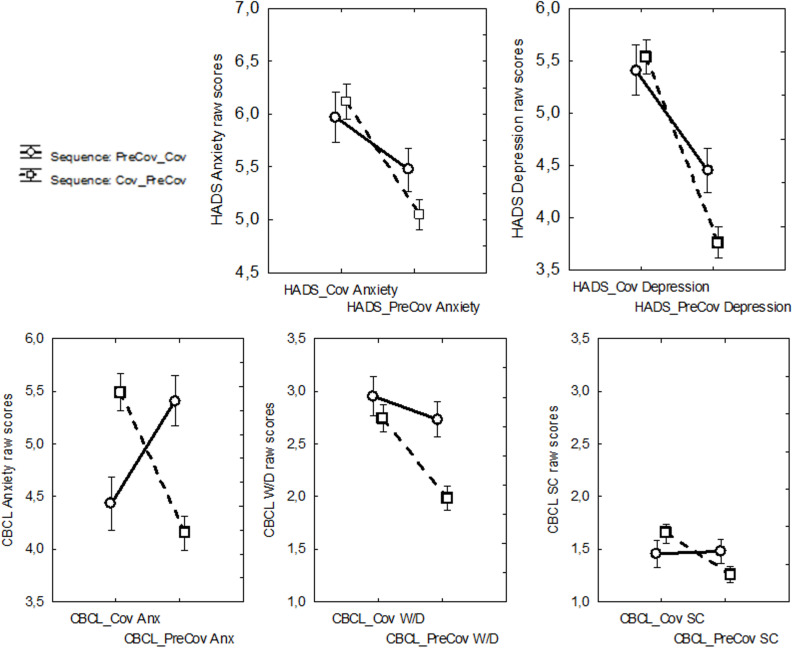
Time (the two repetitions of a questionnaire in the Cov – with reference to the Covid-19 health emergency- and Pre_Cov - with reference to the period preceding the start of the Covid-19 outbreak- conditions) × Sequence (PreCov_Cov and Cov_PreCov) interaction obtained from mixed model repeated-measure ANOVAs for the measures (1) HADS (Hospital Anxiety and Depression) Anxiety, (2) HADS Depression, (3) CBCL (Child Behavior Checklist) Anxiety, (4) CBCL Withdrawn/Depressed, and (5) CBCL Somatic Complaints. W/D, withdrawn/depressed; SC, somatic complaints. Error bars indicate standard errors of the means.

In the following analysis, we directly compared parents’ reported Cov and Pre_Cov data for each HADS and CBCL subscale. For each measure, we ran a mixed model repeated-measure ANOVA considering the between-subject factor of Sequence (Cov_PreCov and PreCov_Cov) and the within-subject factor of Time (i.e., the two repetitions of a questionnaire). For parents’ anxiety and depression symptoms (measured with the two subscales of the HADS questionnaire), both ANOVAs showed a significant main effect of Time [Cov > PreCov; *F*(1,719) = 109.11, *p* < 0.01, $\eta_{\text{p}}^{2}$ = 0.131 and *F*(1,719) = 38.53, *p* < 0.01, $\eta_{\text{p}}^{2}$ = 0.051, respectively for HADS Depression and Anxiety] and a significant Time × Sequence interaction with small effect sizes [*F*(1,719) = 9.69, *p* < 0.01, $\eta_{\text{p}}^{2}$ = 0.013 and *F*(1,719) = 5.13, *p* < 0.03, $\eta_{\text{p}}^{2}$ = 0.007, respectively for HADS Depression and Anxiety]. The interaction was due to the two sequences mainly differing in the PreCov condition in which scores were higher when this condition comes first (Sequence: PreCov_Cov) than second (Sequence: Cov_PreCov) (Table 1 and Figure 2).

Similarly to parents’ HADS data, for the CBCL, the three ANOVAs also returned a significant main effect of Time [Cov > PreCov; *F*(1,719) = 48.01, *p* < 0.01, $\eta_{\text{p}}^{2}$ = 0.062, *F*(1,719) = 3.88, *p* < 0.05, $\eta_{\text{p}}^{2}$ = 0.005 and *F*(1,719) = 15.06, *p* < 0.01, $\eta_{\text{p}}^{2}$ = 0.021, respectively for CBCL Withdrawn/Depressed, Anxiety and Somatic Complaints] and a significant Time × Sequence interaction with small (CBCL Withdrawn/Depressed and CBCL Somatic Complaints) and large (CBCL anxiety) effect sizes [*F*(1,719) = 14.56, *p* < 0.01, $\eta_{\text{p}}^{2}$ = 0.019, *F*(1,719) = 158.70, *p* < 0.01, $\eta_{\text{p}}^{2}$ = 0.180, and *F*(1,719) = 18.70, *p* < 0.01, $\eta_{\text{p}}^{2}$ = 0.025, respectively for CBCL Withdrawn/Depressed, Anxiety and Somatic Complaints]. Similarly to parents’ data, the interactions concerning CBCL Withdrawn/Depressed and Somatic Complaints were due to the two sequences differing in particular in the PreCov condition. By contrast, the effect for the CBCL Anxiety was due to a cross-over interaction as the two sequences also differed in the Cov condition: participants assigned to the Cov_PreCov Sequence rated their children having higher anxiety in the Cov vs. PreCov condition, while participants assigned to the PreCov_Cov Sequence rated their children having higher anxiety in the PreCov vs. Cov condition (see Table 1 and Figure 2).

Overall, the findings obtained from the analyses of variance showed that, in general, internalizing symptoms of parents and children were reported to be significantly higher during the Covid-19 pandemic than before it started. Nonetheless, they also showed that the sequence with which parents had to rate their own anxiety and depression symptoms (and those of their children) significantly influenced their assessments.

## Discussion

The aim of this study was to investigate the immediate impact of the Covid-19 outbreak on families’ mental health. We focused on internalizing symptoms, such as anxiety and depression, of the responding members of the parenting couples who evaluated their own symptoms (through the HADS and the IES-R questionnaires) and those of their children aged between 6 and 18 years (through the CBCL questionnaire). In order to have a self-reported baseline measure of these symptoms, participants had to fill in the questionnaires (HADS and CBCL but not IES-R) twice: once referring to the current health emergency (the Cov condition) and once referring to before it started (the PreCov condition).

The present findings suggest that most parents likely had enough psychosocial resources to respond to the pandemic emergency distress: in fact, present sample’s mean levels of current anxiety, depression and post-traumatic stress did not differ from those of the normal population. Nonetheless, the results also suggest that the pandemic may still have affected the mental health of a considerable number of parents and children, contributing to raise their levels of internalizing problems. First, focusing on the Cov condition, we found that approximately a quarter of the parents reported moderate to severe post-traumatic stress symptoms while about one in 10 showed elevated symptoms of anxiety and depression. As regard parents’ evaluation of children’s internalizing problems, about a quarter of children was rated as having elevated anxiety and depression while about one in ten was rated as having clinically relevant somatic complaints problems. Moreover, internalizing symptoms of parents and children were globally reported to be higher during the Covid-19 pandemic than before it started.

Globally, these data corroborate previous findings highlighting the negative psychological impact of quarantine and lockdown periods, linked to both Covid-19 outbreak and other past health emergencies such as SARS, Ebola, H1N1 influenza pandemic, on mental health symptoms of both adults and children, including post-traumatic stress, depression, anxiety and emotional symptoms (Brooks et al., 2020; Di Giorgio et al., 2020, PREPRINT; Spinelli et al., 2020).

More specifically, the main analyses of the present study focused on the Cov condition and employed a series of multiple linear regression models carried out on parents and children’s anxiety and depression symptoms and on parents’ post-traumatic stress and children’s somatic complaints symptoms. Most importantly, the results showed that to have much fear of being infected by the new coronavirus positively predicted post-traumatic, anxiety, and depression scores of parents. By contrast, having thought often or very often during the emergency (as happened to approximately half of the sample) about possible secondary positive effects or implications of the pandemic, negatively predicted parents’ internalizing and post-traumatic stress symptoms. To continue working and practicing sport during the health emergency also protected parents from internalizing problems (and in particular from depression).

The data on parents’ internalizing symptoms suggest that fear of contagion is an important psychological factor that negatively impacts psychological well-being of healthy adult individuals, subject to isolation and confinement to prevent spread of the new coronavirus. This is in line with results of previous studies showing that fear of infection was a significant stressor during quarantine (see the reviews by Brooks et al., 2020) or, more generally but specifically related to Covid-19, that higher perceived risk of infection increased individuals’ stress and anxiety (Simione and Gnagnarella, 2020). Of interest, in one study investigating the school’s communities response to school closure during the H1N1 2009 influenza pandemic (Braunack-Mayer et al., 2013), it was found that the individuals who were more concerned about becoming infected or spreading the virus to others tended to be those with young children (or to be pregnant women). Unfortunately, in our study we did not ask details about why the participants were afraid of being infected (i.e., if they were afraid of infecting their children or older family members or being infected by them). Nonetheless, it is worth noting that, in our sample, increased levels of parents’ internalizing problems were found in women and in the participants with younger children.

Taken together, our results suggest the need for psychoeducational and psychological support interventions that can reduce excessive fear of contagion in parents, even in those reasonably protected from fatal complications related to the SARS-CoV2 virus (such as the participants in our sample who were without serious clinical conditions and with an average age of about 40 years). Such interventions, which could be delivered online or through smartphone technology, could be designed to make fear manageable and not overwhelming. This could be obtained by means, for example, of cognitive-behavior and mindfulness-based therapies that may, on the one hand, challenge cognitive biases of individuals with exaggerate fear and perception of the risk of being infected and, on the other hand, help individuals to enhance stress management and reduce maladaptive coping strategies such as avoidance and excessive self-criticism (Fischer et al., 2020; Ho et al., 2020; Matiz et al., 2020). The effect of such interventions could therefore help diluting the direct negative effects of fear on the levels of anxiety and stress and depression of individuals, also indirectly affecting the quality of relationships of these people (see Simione and Gnagnarella, 2020 for similar arguments).

Physical activity and the possibility of continuing to work, from home or in the workplace, act as protective factors against the internalizing problems of the parents, especially in favor of depressive symptoms. On the one hand, these data corroborate and extend the results of other research, carried out both within the current health emergency and before it began (e.g., Schuch et al., 2016; Maugeri et al., 2020), demonstrating the positive impact of a physically and intellectually active lifestyle to relieve the symptoms of depression. On the other hand, the present findings suggest the importance of not giving up physical activity even during periods of isolation and social confinement, possibly underlining its importance through targeted psychoeducational and support interventions that, however, should be able to calibrate the right amount of exercise for each individual person, in terms of frequency, duration and intensity of physical activity (Carriedo et al., 2020), also taking into account the possible frustrations that could arise due to the restrictions imposed by the epidemic on physically more active people (Zhang et al., 2020).

What we called “broadening of biased attention” was another important factor that negatively predicted parents’ internalizing problems and post-traumatic stress during the Covid-19 outbreak. This factor reflected the propensity of the parents to think, during the lockdown period, about possible secondary implications of the pandemic, both for their own life and for the environment, and could reveal an emerging element of resilience in the face of adversity (Smith et al., 2020). From this point of view, broadening of biased attention to the crisis by perceiving possible secondary implications can mean not being pervaded and overwhelmed by the uncertainty and stress connected to it; it can rather mean preserving and nurturing a system of meaning, individual but also shared with one’s family, which can bring security and hope during the pandemic. Recent evidence suggests that a crucial aspect of family resilience in response to the Covid-19 pandemic would indeed be the optimization of a system of family beliefs that help the individuals in providing a framework of understating events related to the pandemic (Prime et al., 2020). Accompanying policies to help parents and families during these times of uncertainty will be crucial to identify vulnerability of some households, in order to work to promote paths toward greater capacity for resilience (Prime et al., 2020).

Turning to the regression analyses on children’s internalizing symptoms (as rated by their parents), they primarily highlighted the high and positive predictive value of parents’ current internalizing problems. Children were then evaluated as more depressed and with more symptoms of somatic complaints as their parents had no or little fear of contagion. In other words, the more afraid they were of the infection, the lower the depression and somatic complaints symptoms attributed to their children. These data confirm previously described associations between children and parents’ psychopathological symptoms including depression (e.g., Sellers et al., 2013; Yap et al., 2014). Thus, parents’ experience of symptoms of anxiety and depression during the health emergency was associated with the tendency to attribute similar symptoms to children. Nevertheless, specific symptoms that have to do with the withdrawal, depression and somatization of children seem less recognized by parents having much fear of being infected by the new coronavirus. One hypothesis could be that these parents find themselves excessively involved, and eventually overwhelmed, in their concerns and fear that they could not recognize specific experiences of their children. It is known that being emotionally overwhelmed by situations can compromise the ability to judge oneself, others and events (e.g., Izard, 2002). It is interesting to note that the fear of contagion did not play a predictive role with regard to the anxiety of children that perhaps was more manifest and externalized and thus observable by parents.

In line with these arguments, the direct comparison between parents’ reported Cov and Pre_Cov data as a function of the order with which each questionnaire was completed showed that participants tended to report a smaller difference between Cov and PreCov symptoms of anxiety and depression when they first assessed the latter and then the former symptoms. This may suggest that parents’ report of PreCov anxiety and depression tended to be influenced by the current emergency condition in which participants found themselves while they had to, for the first time (at least in the context of our survey), reflect on their psychological well-being and that of their children.

Beyond these interpretations, it is worth noting that fear of contagion had a different predictive role when parents evaluated themselves with respect to their children, just as the “broadening of biased attention” factor that mediated parents’ internalizing symptoms but not those attributed to children. These data confirm that the perception of a child’s internal experience reported by the parent is founded in a relationship composed of many characteristics both of the child and of the parent, as well as being influenced by factors such as, for example, the purpose of the evaluation and the contingent conditions under which the parent has the opportunity to observe the child (Smith, 2007). It is therefore not surprising that the variables that help explaining parents’ self-perception may diverge, at least in part, from those playing a role in the assessment of children.

The observed findings extend to middle childhood and to internalizing problems previous findings of researches on the psychological sequelae of the Covid-19 pandemic that showed, in children of 4 years of age, an interplay between mothers’ reported difficulties in regulating their own emotions and those of their children in inhibitory self-control (Di Giorgio et al., 2020 PREPRINT). Yet related to Covid-19 outbreak, another study carried out on Italian parents of 2–14 years old children (mean age = 7 years) has recently shown that the impact of lockdown on children’s emotional and behavioral problems was mediated by their parents’ individual and dyadic stress: the higher the parents’ individual and dyadic stress, the more psychological problems children had (Spinelli et al., 2020). A hypothesis was put forward by these authors that lockdown made more difficult for parents to be supportive for their children and this could contribute to the manifestation of their problems. In line with this, previous findings showed that higher levels of anxiety and depression among youth is associated with weaker support from parents (Yap et al., 2014) and that children’s perception of being rejected or accepted by their caregivers is linked to their psychological well-being (Khaleque, 2015).

Taken together, our and previous data indicate the importance of organizing psychological support interventions aimed at families that take into consideration the mental health of parents, but which also take into account the reduced personal resources of the children to face the many changes imposed by the pandemic (Sprang and Silman, 2013). For example, in order to reduce the negative impact of the pandemic on children, it appears necessary to help parents communicate effectively with their children regarding the restrictions imposed by the health emergency (Dalton et al., 2020; Spinelli et al., 2020). Moreover, parents should also be helped so that they can in turn generate hope and instill security in their children as well as, more practically, know how to negotiate family rules, rituals, and routines in the new balance imposed by the pandemic (Prime et al., 2020).

The observed findings need to be interpreted bearing in mind some study limitations that may be addressed by future research. The first limitation concerns the absence of a direct assessment and observation of children and the sole use of self-report measures, which rely on participants’ capabilities to evaluate themselves, as well as being susceptible to desirable responding, acquiescence, and possibly biased by semantic understanding of the scales (Schwarz, 1999). Moreover, particular caution should be given in the interpretation of retrospective questionnaires, collected during emergency periods, before people have been able to restore a sufficient sense of security that may allow them to “decenter” from the current situation, possibly regaining greater clarity and objectivity in the assessments. In our study it is worth noting that we found significant and positive correlations between parents’ current level of post-traumatic stress symptoms (IES-R_Cov) and their current (HADS_Cov total score and CBCL_Cov total Internalizing score), but also past assessments (HADS_PreCov total score and CBCL_PreCov total Internalizing score; all Rho > 0.29, *p* < 0.001).

Second, we compared the mean scores obtained by our sample at the IES-R with non-national normative data. Third, our sample lived in northern and central Italy and these areas were among the most affected by the new coronavirus infections; therefore, we cannot assume that our findings can be generalized to the whole Italian national population. Also, our choice to limit the aims of the present investigation to internalizing problems can be extended in future studies to children’s externalizing behaviors. Of importance, we need to consider that internalizing symptoms, and possibly to a lesser extent externalizing behaviors, attributed to children by parents may be underestimated by them, as suggested by previous research in samples of non-clinical children (Smith, 2007). It would also be important that future longitudinal studies extend the current findings in order to monitor parents and children’s changes in mental health on the basis of the progress of the various phases of the current and any future global health emergencies.

A final issue concerns the limited scope of our analyses, which did not deepen the exploration of possible intervening mechanisms also due to the possibility that the retrospective data collected could have been influenced by the current emergency situation. Future studies using mediation analysis may shed light on the mechanisms underlying the observed relationships between variables, e.g., whether parents’ behaviors during the lockdown had mediated the relationship between parents’ mental health before and during the pandemic as well as their views of their children’s psychological health. Determining the mechanisms that explain the increased rates of internalizing problems will inform the policies used to manage the pandemic to achieve a better balance between infection control and mitigation of negative psychosocial effects (Holmes et al., 2020).

In conclusion, in addition to showing a direct effect of the pandemic on the psychological health of parents and children, the present results also give a series of important information on how parents perceive, and therefore influence, their children in this period of emergency. Our findings thus highlight the urgent need to provide parents with adequate support to take care of their own psychological wellbeing and to help their children coping with the direct and indirect effects of the pandemic.

## Data Availability Statement

The raw data supporting the conclusions of this article will be made available by the authors upon request, without undue reservation.

## Ethics Statement

The studies involving human participants were reviewed and approved by local Ethics of the University of Udine. The patients/participants provided their written informed consent to participate in this study.

## Author Contributions

CC, SF, AM, AP, EV, PC, and FF contributed to conception and design of the study. AM, AP, and CC carried out the investigation. SF and CC organized the database, performed the statistical analysis, and wrote the first draft of the manuscript. All authors contributed to manuscript revision, read, and approved the submitted version.

## Conflict of Interest

The authors declare that the research was conducted in the absence of any commercial or financial relationships that could be construed as a potential conflict of interest.
